# Influence of Pokémon Go on physical activity levels of university players: a cross-sectional study

**DOI:** 10.1186/s12942-017-0080-1

**Published:** 2017-02-22

**Authors:** Fiona Y. Wong

**Affiliations:** Faculty of Health and Social Sciences, The Hong Kong Polytechnic University, Kowloon, Hong Kong China

**Keywords:** Physical activity levels, Pokémon Go, Active lifestyle, Augmented reality games

## Abstract

**Background:**

The prevalence of overweight is increasing and the effectiveness of various weight management and exercise programs varied. An augmented reality smartphone game, Pokémon Go, appears to increase activity levels of players. This study assessed the players and ex-players’ frequencies and durations of staying outdoors, and walking/jogging before and during the time they played Pokémon Go, evaluated the physical activity levels of players, ex-players and non-players, and investigated the potential factors which determined their play statuses.

**Methods:**

Students in a university answered an online-questionnaire survey. The IPAQ-short form was incorporated to measure vigorous-intensity activities, moderate-intensity activities and walking. Chi square tests were used to compare frequencies and durations of staying outdoors and walking/jogging, health discomforts and physical activity levels between players, ex-players and non-players. Wilcoxon signed ranks tests were performed to assess the changes prior to and during the time when the players and ex-players played Pokémon Go. Logistic regression analyses were performed to assess factors contributing to playing, quitting or not playing Pokémon Go.

**Results:**

644 university students answered the questionnaire. Compared with the ex-players, the players were significantly more frequent to stay outdoors when playing Pokémon Go (P < 0.001), walk/jog to a location to catch Pokémon, to Pokéstops or Gyms (P < 0.005), as well as walking/jogging to hatch eggs (P < 0.001). The players spent a mean of 108.19 ± 158.21 min/week to walk/jog in order to play the game which is equivalent to burning 357 kcal/week for a 60-kg person walking a moderate pace. Compared with the non-players, players were more likely to be aged 18–25 years [OR (95% CI) 3.28 (1.28–8.40), P = 0.013], never [OR (95% CI) 10.51 (1.12–98.57), P = 0.039] or rarely [OR (95% CI) 4.00 (1.95–8.23), P < 0.001] stayed outdoors and rarely walked/jogged prior to playing the game [OR (95% CI) 3.88 (1.86–8.05), P < 0.001]. However, there was no significant difference in physical activity levels between the three groups (P = 0.573).

**Conclusions:**

Players who used to be sedentary benefited the most from Pokémon Go. The game can be used as a starting point for sedentary people to begin an active lifestyle. The impact of Pokémon Go on physical activity can provide insights to public health workers in using novel strategies in health promotion.

## Background

Sedentary lifestyle and physical inactivity are associated with obesity and chronic illness. According to WHO, 60–85% of people in the world lead a sedentary lifestyle. A sedentary lifestyle doubles the risk of cardiovascular diseases, diabetes and obesity, and increases the risks of colon cancer, high blood pressure, lipid disorders and depression [1]. Similar to populations in many countries, people in Hong Kong tend to have less physical activities. According to the statistics of the Centre for Health Protection collected in April 2014, 20.3% of the Hong Kong people had low physical activity level [2]. Compared with the same survey conducted in Oct 2005 in which 19.7% were categorized as having low physical activity, this indicates that there has been no significant improvement in people’s physical activity level regardless of the exercise promotion programs delivered in the past 10 years [3]. WHO recommends adults aged 18–64 to have at least 150 min of moderate-intensity aerobic physical activity throughout the week, or at least 75 min of vigorous intensity physical activity a week, or an equivalent combination of moderate and vigorous physical activity [4]. However, in Hong Kong only 37.4% of the people surveyed met the WHO recommendation in 2014 [5].

In the past few decades, weight management programs primarily using dietary and exercise interventions had been developed and delivered to help people to achieve desirable anthropometric and metabolic outcomes. However, the effects of these programs varied [1, 6]. The prevalence of overweight and obesity is increasing regardless of the hard work of the health workers. In the US, the prevalence of overweight of people aged 18 years or above increased from 70.3% in 2010 to 72.1% in 2014 while in China, the prevalence rate increased from 31.1 to 36.2% [7]. As smartphones and mobile games are becoming more popular, and it seems that there are limitations in the traditional approaches in promoting exercises and physical activities, mobile exergames (mobile games which combine exercising with game play) could be another option to increase physical activity levels in daily life [8]. The potential for smartphone applications to facilitate health behavioral change has been supported [9, 10]. Some mobile phone games for health behaviors have been tested for their short-term effectiveness in promoting physical activity and healthy eating [11, 12]. The most popular smartphone applications are found to be those incorporated with virtual avatars, gaming and social media [12]. The self-determination theory (SDT), a motivational theory, has been used in examining participation in digital games [13–15]. SDT explains the relationships between motives, psychological needs, motivations and outcomes [16]. The three basic psychological needs—competence (capable and challenged), autonomy (volitional and controllable) and relatedness (sense of belongingness) are essential for people’s well-being and psychological growth. Games with virtual avatars are generally higher in autonomy satisfaction as they allow players to develop their own in-game characters and engage in any activities they want. Gaining points, rewards and prestige in games increase motivations as the players feel more competent. Interactions between players can facilitate relatedness needs. The latest augmented reality (AR) smartphone game, Pokémon Go, which has been downloaded by millions of people since its launch, seems to be able to satisfy the psychological needs in SDT.

Pokémon Go is an AR game combining real-world and virtual elements. The mission is to capture all Pokémon creatures appear on the screen [17]. First the player needs to create his on-screen avatar, the trainer. Using the global positioning system (GPS), the smartphone will vibrate to inform the player that a Pokémon is nearby as he moves around. The player then needs to throw a Pokéball to catch it. The game uses a real-world map of the streets and landmarks to help players to locate Pokémon, Pokéstops (for collecting Pokéballs and other special items) and Gyms (for putting the Pokémon in a battle with other players’ Pokémon). Players can play Pokémon Go indoors if they come close to a Pokéstop. However, if players want to level up quickly and collect as many Pokémon as possible, especially those rare ones, they need to be active. They need to move around outdoors in real life to look for Pokémon, go to different Pokéstops and Gyms, as well as walking/jogging for a certain distance in order to hatch eggs. Some studies have found that Pokémon Go has the potential to increase short-term activity [18–21], and provide emotional benefits to players [22, 23].

Since the launch of Pokémon Go, it has become one of popular game applications around the world [24, 25]. The game was released in Hong Kong on 25 July 2016. It is easy to find people, especially young adults, playing Pokémon Go on the streets. Unlike most of the computer and video games which require players to play indoors, Pokémon trainers need to be physically active. Well-designed games played on mobile phones have the potential to encourage voluntary physical activity. The aim of this study is to investigate the impact of Pokémon Go on physical activity of student players in a local university. The objectives are (1) to compare the players and ex-players’ frequencies and durations of staying outdoors, and walking/jogging before and during the time they played Pokémon Go; (2) to assess and compare the physical activity levels, including vigorous activities, moderate activities and walking, of players, ex-players and non-players of Pokémon Go; and (3) to investigate the potential factors which determine play statuses of the university students.

## Methods

### Setting and target group

This is a cross-sectional study using an online-questionnaire survey approach. An invitation email was sent to all students in a local university. Those who refused to receive commercial electronic messages (CEM) were excluded. According to the email system of the university, a total of 21,935 students were willing to receive CEM from the university. A sample of 378 students would provide a margin of error of 5% from the true values at 95% confidence level.

The survey was conducted 28 days after the game was released in Hong Kong. An invitation email explaining the purpose of the survey was sent to all students who agreed to receive CEM on 22 Aug 2016. Two hyperlinks, one for accessing the Chinese online questionnaire and another one for accessing the English online questionnaire, were enclosed in the email. The online-questionnaire survey ended on 5 Sep 2016.

### Measuring instrument

The questionnaire consisted of 31 closed-ended questions in the format of multiple choice and Likert-scale. Depending on the play statuses of the participants, the questions they needed to answer varied. The questions measured time spent in playing Pokémon Go, frequencies and time spent in staying outdoors, walking and/or jogging intentionally when playing the game and before started playing the game, and health discomforts experienced. Characteristics of the respondents including sex, age, academic program attending and employment status were also studied.

The International Physical Activity Questionnaire (IPAQ)—short form has been validated and translated into different languages, including Chinese, was used to measure three specific types of activity, including vigorous-intensity activities, moderate-intensity activities and walking, in the past seven days [26]. Metabolic Equivalent of Task (MET) is the energy cost of physical activities. The three activities have different MET values [vigorous physical activity (PA) = 8.0 METs; moderate PA = 4.0 METs and walking = 3.3 METs]. The separate scores on vigorous-intensity activities, moderate-intensity activities and walking, and a combined total PA score were calculated and expressed in MET-min/week [27]. Physical activity levels were categorized as low, moderate and high based on the total PA and the criteria listed in the scoring protocol.

The questionnaire was pilot-tested online with 16 subjects aged 18–40 years. Eleven were players, three were ex-players and two were non-players. Among the players, six (54.5%) spent 60 min or less a day to play Pokémon Go, eight (72.7%) reported that they stayed outdoors sometimes or most of the time and four (36.4%) claimed that they stayed outdoors at least an hour a day intentionally to play the game. Regarding walking/jogging when playing Pokémon Go, seven (63.6%) of them would walk/jog to specific locations to catch Pokémon, to reach Pokéstops or Gyms sometimes or most of the time, and five (45.5%) would walk/jog for 10–30 min a day. Six players (54.5%) claimed that that would walk/jog sometimes or most of the time in order to hatch eggs. The questionnaire was appropriately adjusted before implementation of the main study. The response choices of those questions regarding frequencies and durations in staying outdoors, walking/jogging were revised. To facilitate the respondents in completing the questionnaire online, the wordings and formats of some of the questions were also revised.

### Statistical analysis

Statistical analysis was performed using SPSS (version 21). Descriptive statistics were reported by mean ± standard deviation or percentage, as appropriate. Chi square tests were used to compare frequencies and durations of staying outdoors and walking/jogging, health discomforts and physical activity levels between players, ex-players and non-players. Wilcoxon signed ranks tests were performed to assess the changes in frequencies in staying outdoors and walking/jogging prior to and during the time they played Pokémon Go. Univariate analyses, followed by logistic regression analyses were performed to assess socio-demographic factors and play behaviors which determined play statuses. A P value of <0.05 was considered as statistically significant.

## Results

### Characteristics of the subjects

A total of 784 students responded to the online questionnaire. Among these 784 subjects, 644 completed the questionnaire. 39.5% claimed that they were players (still playing Pokémon Go at the time of the survey), 30.9% claimed that they were ex-players (played before but had not played for at least 7 consecutive days), and the remaining 29.6% were non-players (never played the game) (Table 1). 48.2% of these respondents were male, and the majority were 18–25 years old (72.5%) and currently enrolling in the full-time bachelor’s degree programs (70.1%) in the University. Approximately half of them were not employed (49.1%).

**Table 1:** Play status and socio-demographics of subjects

	N = 644 N (%)
Play status	
Players	243 (37.7%)
Ex-players	178 (27.6%)
Non-players	223 (34.6%)
Sex	
Male	275 (48.2%)
Female	296 (51.8%)
Missing/reject	73
Age (years)	
Under 18	75 (13.1%)
18–25	416 (72.5%)
26–30	46 (8.0%)
31–40	29 (5.1%)
41–50	6 (1.0%)
51–60	2 (0.3%)
Missing/reject	70
Academic program attending	
Full-time bachelor’s degree	387 (70.1%)
Full-time postgraduate degree	85 (15.4%)
Part-time postgraduate degree	44 (8.0%)
Higher diploma	36 (6.5%)
Missing/reject	92
Employment status	
Not working	277 (49.1%)
Working full-time	95 (16.8%)
Working part-time	192 (34.0%)
Missing/reject	80

### Play behaviors of players and ex-players

Play behaviors of players and ex-players were compared. For players, questions focused on their behaviors in the past seven days. For ex-players, questions focused on the period they were still playing Pokémon Go. Players played 4.79 days (SD = 2.11) a week which was significantly longer compared with the ex-players who played 3.57 days (SD = 2.26) a week (P < 0.001) but there was no significant difference in their time spent on the game each day (Table 2). Approximately 60 and 62% of the players and ex-players, respectively, spent less than one hour a day on the game (P = 0.274).

**Table 2:** Play behaviors and health discomforts between players and ex-players

	Players group	Ex-players Group	Chi-square	P
	N (%)	N (%)	χ^2^	
Time spent each day in playing Pokémon Go				
≤15 min	37 (15.2%)	40 (22.5%)	7.54	0.274
16–30 min	48 (19.8%)	40 (22.5%)		
31–60 min	61 (25.2%)	30 (16.9%)		
>60 min to 2 h	46 (18.9%)	30 (16.9%)		
2–3 h	26 (10.7%)	16 (9.0%)		
3–4 h	14 (5.8%)	13 (7.3%)		
≥4 h	11 (4.5%)	9 (5.1%)		
Frequency in staying outdoors to play Pokémon Go				
Never	50 (20.6%)	51 (28.7%)	20.43	<0.001
Rarely	69 (28.4%)	75 (42.1%)		
Sometimes	100 (41.2%)	40 (22.5%)		
Most of the time	24 (9.9%)	12 (6.7%)		
Time spent each day in staying outdoors to play Pokémon Go				
0 min	50 (20.6%)	51 (28.7%)	9.34	0.096
≤15 min	37 (15.2%)	24 (13.5%)		
16–30 min	42 (17.3%)	33 (18.5%)		
31 – 60 min	52 (21.4%)	21 (11.8%)		
>60 min – 2 hr	35 (14.4%)	24 (13.5%)		
≥2 h	27 (11.1%)	25 (14.0%)		
Frequency in walking/ jogging to catch Pokémon, go to Pokéstops or Gyms				
Never	67 (27.6%)	70 (39.3%)	13.06	0.005
Rarely	72 (29.6%)	60 (33.75)		
Sometimes	80 (32.9%)	33 (18.5%)		
Most of the time	24 (9.9%)	15 (8.4%)		
Time spent each day in walking/ jogging to catch Pokémon, go to Pokéstops or Gyms				
0 min	67 (27.6%)	70 (39.3%)	11.26	0.046
<10 min	52 (21.4%)	36 (20.2%)		
10–20 min	48 (19.8%)	34 (19.1%)		
21–30 min	37 (15.2%)	13 (7.3%)		
31–60 min	24 (9.9%)	12 (6.7%)		
≥1 h	15 (6.2%)	13 (7.3%)		
Frequency in walking/jogging to hatch eggs				
Never	92 (37.9%)	99 (55.6%)	22.09	<0.001
Rarely	76 (31.3%)	56 (31.5%)		
Sometimes	58 (23.9%)	20 (11.2%)		
Most of the time	17 (7.0%)	3 (1.7%)		
Health discomforts				
Eye strain	28 (11.5%)	22 (12.4%)	0.07	0.879
Neck pain	22 (9.1%)	11 (6.2%)	1.17	0.360
Shoulder pain	18 (7.4%)	13 (7.3%)	0.002	1.000
Wrist pain	10 (4.1%)	2 (1.1%)	3.32	0.080
Finger fatigue	18 (7.4%)	7 (3.9%)	2.22	0.150
Leg pain/tiredness	30 (12.3%)	7 (3.9%)	9.07	0.003
Mental tiredness/weariness	15 (6.2%)	15 (8.4%)	0.79	0.444
None	164 (67.5%)	136 (76.4%)	3.99	0.050
Game continuation^a^				
Few days—a week	18 (7.7%)	NA		
2–3 weeks	19 (8.1%)			
1 month	20 (8.5%)			
2–3 months	23 (9.9%)			
4–12 months	16 (6.8%)			
>I year	28 (12.0%)			
Don’t know	110 (47.0%)			
*Missing*	9			

	T-test		P	
	Players Group	Ex-players group		
	Mean ± SD	Mean ± SD		
Estimated time spent in walking/ jogging to catch Pokémon, go to Pokéstops or Gyms (min/week) All respondents in the group	98.43 ± 151.21	68.23 ± 141.82	0.038	

	Players Group		Ex-players group	
	Mean ± SD	P	Mean ± SD	P
Prior walking/jogging frequencies				
Never/rarely	108.19 ± 158.21	0.351	70.39 ± 148.25	0.838
Sometimes/most of the time	89.88 ± 145.43		66.02 ± 135.73	

Refers to play behaviors of players in the last 7 days, and play behaviors of ex-players when they were still playing Pokémon Go

^a^The question on game continuation is for players only

Compared with the ex-players group, the players group was significantly more frequent to stay outdoors when playing Pokémon Go, walk/jog to locations to catch Pokémon, to Pokéstops or Gyms, as well as walking/jogging in order to hatch eggs (Table 2). Around half of the respondents in the players group (51.1%) stayed outdoors sometimes or most of the time to play the game but only 29.2% in the ex-players group would do the same (P < 0.001). Compared with the players group, more ex-players (28.7 vs 20.6%) claimed that they never stayed outdoors to play Pokémon Go but less ex-players (43.8 vs 53.9%) claimed that they spent at least 60 min a day in outdoor to play the game, however, the differences were insignificant (P = 0.096). On the other hand, 42.8% of the players group reported that they walked/jogged to specific locations to catch Pokémon, to Pokéstops or Gyms sometimes or most of the time but only 26.9% of the ex-players group would do the same (P = 0.005). 31.3% of the players walked/jogged for more than 20 min a day to catch Pokémon, to Pokéstops or Gyms, which was also significantly more than the ex-players (21.3%) (P = 0.046). In order to hatch eggs, 30.9% of the players would walk/jog sometimes or most of the time, but 87.1% of the ex-players claimed that they never or rarely walked/jogged to hatch eggs (P < 0.001).

We also estimated the time the players and ex-players spent in a week to walk/jog intentionally to play Pokémon Go by multiplying the reported number of days they played the game in a week and the length of time they walked/jogged to catch Pokémon. The players spent a mean of 98.43 ± 151.21 min/week to walk/jog intentionally which was significantly longer than the ex-players (68.23 ± 141.82 min/week) (P = 0.038) (Table 2). According to their frequencies of walking/jogging prior to playing the game, the players and ex-players were separated into two groups. One group was those who claimed that they never or rarely walked/jogged before playing Pokémon Go and another group was those who used to walk/jog sometimes or most of the time. Among the players, those who claimed that they never or rarely walked/jogged before spent a longer time each week in walking/jogging when playing the game (108.19 ± 158.21 min/week) compared with those players who used to walk/jog sometimes or most of the time (89.88 ± 145.43 min/week), however the difference was insignificant (P = 0.351). Insignificant difference was also found among the ex-players (P = 0.838).

Regarding health discomforts, the players (12.3%) reported significantly more leg pain or tiredness than the ex-players (3.9%) (X^2^ = 9.07, P = 0.003) (Table 2). A significantly higher proportion of respondents in the ex-players group (76.4 vs 67.5%) reported that they did not have any health discomforts associated with playing Pokémon Go (X^2^ = 3.99, P = 0.05). When the players were asked to estimate how long they would continue playing the game, 12.0% stated that they would continue playing the game for more than a year, however, 47% had no idea how long they would play (Table 2). The non-players were asked why they did not play the game, the majority stated that they were not interested or did not have time.

### Compare frequencies of staying outdoors and walking/jogging prior to and during playing Pokémon Go

To understand whether Pokémon Go can encourage players to increase physical activity levels, changes in frequencies of staying outdoors and walking/jogging prior to and during the time playing Pokémon Go were assessed using Wilcoxon signed ranks tests. No significant change was found in both the players group and the ex-players groups. However, if only those who claimed that they never or rarely stayed outdoors or walked/jogged prior to playing Pokémon Go were selected for the analyses, positive changes were found.

Regarding staying outdoors, positive ranks indicated that the respondents “stayed outdoors more often to play Pokémon Go” and negative ranks indicated that they “stayed outdoors less often to play Pokémon Go”, compared with the frequencies before playing the game. A positive sum of ranks of 3457.50 and a negative sum of ranks of 637.50 showed that players who never or rarely stayed outdoors prior to playing Pokémon Go, had significantly changed to stay outdoors more often to play the game (Z = −6.11, P < 0.001) (Table 3). Similarly, those who claimed that they never or rarely walked/jogged before playing Pokémon Go, had significantly changed to walk/jog more often to catch Pokémon, to reach Pokéstops or Gyms (Z = −4.34, P < 0.001), and had significantly changed to walk/jog more often in order to hatch eggs (Z = −2.72, P = 0.007).

**Table 3:** Compare the change of frequency of players and ex-players who *never or rarely* staying outdoors and walking/jogging before playing Pokémon Go

	N	Mean rank	Sum of ranks	Z	P
*Players group*					
Frequency in staying outdoors when playing Pokémon Go (–) Before playing Pokémon Go					
Negative ranks	17	37.50	637.50	−6.11	<0.001
Positive ranks	73	47.36	3457.50		
Ties	37				
Frequency in walking/jogging to catch Pokémon, to reach Pokéstops/Gyms (–) Before playing Pokémon Go					
Negative ranks	26	36.00	936.00	−4.34	<0.001
Positive ranks	60	46.75	2805.00		
Ties	31				
Frequency in walking/jogging to hatch eggs (–) Before playing Pokémon Go					
Negative ranks	34	37.00	1258.00	−2.72	0.007
Positive ranks	51	47.00	2397.00		
Ties	32				
*Ex-players group*					
Frequency in staying outdoors when playing Pokémon Go (–) Before playing Pokémon Go					
Negative ranks	12	18.00	216.00	−3.02	0.003
Positive ranks	29	22.24	645.00		
Ties	45				
Frequency in walking/jogging to catch Pokémon, to reach Pokéstops/Gyms (–) Before playing Pokémon Go					
Negative ranks	30	24.00	720.00	−0.69	0.491
Positive ranks	26	33.69	876.00		
Ties	34				
Frequency in walking/jogging to hatch eggs (–) Before playing Pokémon Go					
Negative ranks	40	27.00	1080.00	−3.31	0.001
Positive ranks	14	28.93	405.00		
Ties	36				

For ex-players who never or rarely stayed outdoors prior to playing the game, had significant changed to stay outdoors more often to play Pokémon Go (Z = −3.02, P = 0.003). A significant negative change was found in hatching eggs which indicated that those ex-players who never or rarely walked/jogged before, significantly walked/jogged less often to hatch eggs (Z = −3.31, P = 0.001). No positive change was found in both players and ex-players who claimed that they used to stay outdoors or walk/jog sometimes or most of the time prior to playing the game.

### Factors determine playing or not playing Pokémon Go

Chi square analyses were performed to assess the relationships between play statuses and demographic characteristics, physical activity levels determined by IPAQ and frequencies of staying outdoors and walking/jogging prior to playing Pokémon Go. The three different play statuses were found to have significant relationships with age, employment status and frequencies of staying outdoors and walking/jogging prior to playing the game.

A multinomial logistic regression was used to further assess factors which determined the play statuses (Table 4). Compared with the non-players, players were more likely to be aged 18–25 years [OR (95% CI) 3.28 (1.28–8.40), P = 0.013], never [OR (95% CI) 10.51 (1.12–98.57), P = 0.039] or rarely [OR (95% CI) 4.00 (1.95–8.23), P < 0.001] stayed outdoors and rarely walked/jogged prior to the game [OR (95% CI) 3.88 (1.86–8.05), P < 0.001]. Similarly, compared with the non-players, the ex-players were more likely to be aged <18 [OR (95% CI) 4.05 (1.13–14.56), P = 0.032] or 18–25 [OR (95% CI) 3.17 (1.03–9.72), P = 0.044], never [OR (95% CI) 17.57 (1.81–170.64), P = 0.013] or rarely [OR (95% CI) 3.34 (1.53–7.28), P = 0.002] stayed outdoors and rarely walked/jogged prior to the game [OR (95% CI) 3.62 (1.67–7.87), P = 0.001].

**Table 4:** Factors determine play statuses of Pokémon Go using multinomial logistic regression

	Players OR (95% CI)	P	Ex-players OR (95% CI)	P
Age (years)				
<18	2.94 (0.95–9.14)	0.062	4.05 (1.13–14.56)	0.032
18–25	3.28 (1.28–8.40)	0.013	3.17 (1.03–9.72)	0.044
26–30	2.34 (0.79–6.94)	0.125	1.43 (0.37–5.58)	0.604
≥31	1.00		1.00	
Employment status				
Not working	0.97 (0.59–1.59)	0.902	1.16 (0.70–1.95)	0.565
Working full-time	0.78 (0.39–1.57)	0.483	0.51 (0.22–1.17)	0.114
Working part-time	1.00		1.00	
Staying outdoors prior to playing Pokémon Go				
Never	10.51 (1.12–98.57)	0.039	17.57 (1.81–170.64)	0.013
Rarely	4.00 (1.95–8.23)	<0.001	3.34 (1.53–7.28)	0.002
Sometimes	1.56 (0.83–2.91)	0.164	1.57 (0.80–3.09)	0.188
Most of the time	1.00		1.00	
Walking/jogging prior to playing Pokémon Go				
Never	3.64 (0.84–15.80)	0.085	1.93 (0.39–9.56)	0.420
Rarely	3.88 (1.86–8.05)	<0.001	3.62 (1.67–7.87)	0.001
Sometimes	1.72 (0.93–3.19)	0.084	1.34 (0.69–2.61)	0.394
Most of the time	1.00		1.00	

Reference category: non-players

To study game enjoyment factors which determined the students continued playing or quitted the game for at least seven consecutive days, Chi square tests were performed to assess the relationships between play statuses (players and ex-players) and number of days played in a week, frequencies of and time spent in staying outdoors and walking/jogging when playing Pokémon Go, and health discomforts. Significant relationships were found in number of days played in a week, frequencies of staying outdoors, walking/jogging to catch Pokémon, to reach Pokéstops/Gyms and walking/jogging to hatch eggs, time spent in walking/jogging to catch Pokémon, to reach Pokéstops/Gyms, and leg pain/tiredness.

A binary logistic regression was used to further assess factors which determined the students continued playing (players) or quitted the game (ex-players) (Table 5). Those who played at least 4 days a week [OR (95% CI) 1.87 (1.21–2.90), P = 0.005] and walked/jogged sometimes [OR (95% CI) 2.25 (1.05–4.83), P = 0.038] or most of the time [OR (95% CI) 6.62 (1.54–28.53), P = 0.011] in order to hatch eggs, were likely to continue playing Pokémon Go (Table 5).

**Table 5:** Factors determine whether respondents continued playing Pokémon Go using binary logistic regression

	Players OR (95% CI)	P
Nos of days played/week (days)		
1–3	1.00	
4–7	1.87 (1.21–2.90)	0.005
Frequency in staying outdoors to play Pokémon Go in the last 7 days		
Never	1.00	
Rarely	0.74 (0.40–1.39)	0.350
Sometimes	1.57 (0.70–3.55)	0.277
Most of the time	1.48 (0.44–4.98)	0.530
Frequency in walking/jogging to catch Pokémon, go to a Pokéstop or a Gym		
Never	1.00	
Rarely	0.11 (0.01–1.94)	0.132
Sometimes	0.12 (0.01–2.17)	0.152
Most of the time	0.08 (0.004–1.47)	0.088
Time spent each day in walking/jogging to catch Pokémon, go to a Pokéstop or a Gym		
0 min	1.00	
<10 min	10.30 (0.60–175.79)	0.107
10–20 min	5.95 (0.36–98.36)	0.212
21–30 min	8.95 (0.52–153.15)	0.130
31–60 min	5.35 (0.31–92.09)	0.248
1–2 h	3.19 (0.19–53.93)	0.421
Frequency in walking/jogging to hatch eggs		
Never	1.00	
Rarely	1.53 (0.87–2.67)	0.140
Sometimes	2.25 (1.05–4.83)	0.038
Most of the time	6.62 (1.54–28.53)	0.011
Leg pain/tiredness		
Yes	1.00	
No	0.43 (0.17–1.08)	0.071

### Physical activity levels

**Fig. 1:**
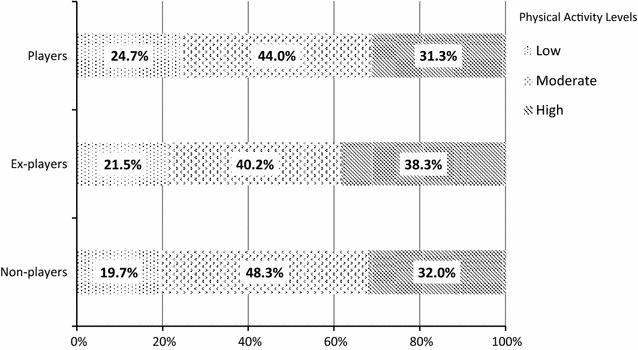
Physical activity levels of players, ex-players and non-players

In addition to play behaviors, physical activity levels in the past seven days were also assessed using IPAQ. A total of 420 subjects, including 166 players, 107 ex-players and 147 non-players, completed this assessment. There was no significant difference in physical activity levels between the three groups (P = 0.573) (Table 6). Around 40–48% of these student respondents were having moderate physical activity level while 31–38% was having high physical activity level (Fig. 1).

**Table 6:** Physical activity levels of players, ex-players and non-players using Chi square

	Players	Ex-players	Non-players	X^2^	P
Physical activity levels					
Low	41 (24.7%)	23 (21.5%)	29 (19.7%)	2.91	0.573
Moderate	73 (44.0%)	43 (40.2%)	71 (48.3%)		
High	52 (31.3%)	41 (38.3%)	47 (32.0%)

Since the ex-players had not played the game for at least seven consecutive days at the time of the survey, they were combined with the non-players to compare walking, moderate, vigorous and total PA with the players using T-tests. The players were found to have higher walking and moderate PA, however, these were not statistically significant (Table 7).

**Table 7:** Walking PA, Moderate PA, Vigorous PA and Total PA of Players and Ex-players/Non-players Using T-tests

	Players Mean ± SD	Ex-players/non-players Mean ± SD	P
Walking PA (MET-min/week)	1437.02 ± 1959.75	1346.61 ± 1740.48	0.579
Moderate PA (MET-min/week)	443.66 ± 1184.71	433.52 ± 1178.05	0.928
Vigorous PA (MET-min/week)	678.04 ± 1313.33	866.95 ± 1750.31	0.187
Total PA (MET-min/week)	2508.83 ± 2947.37	2549.72 ± 3097.30	0.893

## Discussion

This is the first study assessing the play behaviors of Pokémon Go and the physical activity levels of university students in Hong Kong. Our results showed that players who were used to being sedentary benefited the most from Pokémon Go. Players who never or rarely stayed outdoors or walked/jogged before would significantly stay outdoors more frequently as well as walking/jogging more often in order to play Pokémon Go. The players group walked/jogged 98.43 min a week intentionally for playing Pokémon Go. Since walking a moderate pace of 4.8 km/h is estimated to burn 3.3 kcal/kg/h [28], walking 98.43 min/week is equivalent to burning 325 kcal a week for a 60-kg person walking a moderate pace. Most importantly, those players who used to be more sedentary (i.e. never or rarely walked/jogged prior to playing Pokémon Go), walked/jogged 108.19 min a week just for playing Pokémon Go, about 10 min more than the mean of the whole players group. Walking 108.19 min/week is equivalent to burning 357 kcal a week. However, the length of time in walking/jogging prior to playing the game was not studied because of the possibility of recall bias, therefore, the changes in walking time and calorie expenditure were uncertain. A recent study showed that Pokémon Go players walked 1473 steps more a day in average, a 25% increase compared to prior activity level [18]. Another study also found that Pokémon Go users increased their daily average steps by 955 additional steps during the first week of installation although the number of daily steps had gone back to pre-installation levels by the sixth week [19]. From the IPAQ assessment, our players were found to have higher mean walking PA compared with the ex-players and non-players although the difference was insignificant. The higher walking PA in the players group could be attributed to the playing of Pokémon Go.

Players did not spend more time each day to play Pokémon Go compared with the ex-players, but they were more willing to play the game outdoor. The players were also more eager and active when playing. They were more frequent and spent longer period of time to walk or jog to catch Pokémon, to Pokéstops to get more Pokéballs and special items, to Gyms to fight battles, and to hatch eggs in order to get new Pokémon. Although there was no significant difference in the overall physical activity levels and total PA between players, ex-players and non-players, the impact of Pokémon Go on walking or jogging was important, especially to those players who never or rarely walked or jogged before playing the game. Pokémon Go could motivate players who were previously sedentary to become more physically active. As previously discussed, if they did not play Pokémon Go, they were likely to walk 108.19 min less a week. This finding was in line with Althoff’s study of which they found that Pokémon Go players who used to be very inactive exhibited large activity increases [18]. Getting inactive people to do a little bit of physical activity is believed to provide greater population health gains even the goal of 150 min a week cannot be achieved [29]. Participation in leisure time activity, even below the recommended level, is associated with a reduced risk of mortality and longer life expectancy [30], as well as decreasing lifetime medical expenditure [31].

Significant positive increases in frequencies of staying outdoors and walking/jogging could not be observed in players who were relatively more active prior to playing Pokémon Go. Outdoor activities, walking or jogging could be already part of the regular daily activities of these players, therefore, they did not need to spend extra time to walk or jog outdoors intentionally as the players who used to be less active. Ex-players who never or rarely stayed outdoors before were found to stay outdoors more often to play the game, however, they did not walk or jog more often in order to catch Pokémon, to go to Pokéstops or Gyms, or to hatch eggs. Ex-players could be less interested in or enthusiastic about playing Pokémon Go, so they put less effort and quit finally. This was consistent with their significantly lower frequencies in walking/jogging when playing Pokémon Go, compared with the players.

In addition, we found that university students aged 18–25 years, who never or rarely stayed outdoors before and rarely walked/jogged before were more likely to play Pokémon Go, compared with students who were non-players. When we asked the non-players why they did not play the game, the majority stated that they were not interested or did not have time. The overall physical activity levels were determined by the total PA obtained from IPAQ which assessed a combination of walking, moderate and vigorous PA in the past seven days. In this study, there was no significant difference in the overall physical activity levels and total PA between players, ex-players and non-players. The non-players had the highest proportion of people (48.3%) having a moderate physical activity level and the lowest proportion of people having a low physical activity level (19.7%). The reasons behind were uncertain but it seemed that many non-players might participate in regular moderate exercise and they were not interested in Pokémon Go. Sometimes self-reported physical activity could be unreliable because of the possibility of recall bias and reporting bias by social desirability [12]. Some non-players could over-report their activity levels as they did not want to leave the impression that they did not play Pokémon Go because they were lazy to move. Further in-depth assessment is needed to collect more objective data on the types and durations of their exercise. The players group was found to have a higher walking PA than the ex-players/non-players group although there was no significant difference. Part of the walking PA could be attributed to the playing of Pokémon Go. In this study, walking, moderate and vigorous PA prior to playing Pokémon Go were not measured, however, a previous study found that playing Pokémon GO increased moderate to vigorous physical activity by about 50 min per week and reduced sedentary behavior by about 30 min per day [21].

According to the self-determination theory (SDT), autonomy, competence and relatedness were positively associated with enjoyment and preference for future play [15]. In Pokémon Go, an individual can take full control of the movement of his avatar. Collecting different Pokémon may provide people with optimal challenges and a feeling of success. Players can earn rewards every time they catch a Pokémon, put in effort to walk to a Pokéstop, as well as walking or jogging for a certain distance. With regard to relatedness needs, they can fight a battle with other players’ Pokémon, and there are social media groups for sharing play strategies and locations where the rare Pokémon appear. It seems that Pokémon Go can satisfy the three psychological needs in SDT which are responsible for motivating people to continue playing the game; but 47% of the players had no idea how long they would continue playing the game.

One of the challenges of using computer and mobile games to promote health behaviors is that the game enthusiasm usually decreases with time [12]. Pokémon Go is still one of the most downloaded game apps in Apple’s free downloads category three months after its release. However, it is possible that players will stop playing or play less often a few months later if they have collected all the Pokémon. A study found that instant enjoyment, exploration intention (exploration of the game environment) and attention demand (focus on the game) were predictors of interest of game players [32]. Enjoyment of the game was found to increase Pokémon Go players’ physical activities outdoors [33]. Games with an internal social network which allows users to interact, post updates, spread content and comments on others’ posts can help users to remain active and less likely to drop out of the games [34]. The study of the influence of social networks on user behavior in a physical activity tracking application found that the social network increased user offline real-world physical activity by 7% [34]. Researchers need to further explore motivators and appealing features and characteristics of mobile games for sustaining people to continue playing the game or at least for a longer period of time. Although an increase in physical activity could be found in some exergame players but many were short-term impact and there was no strong evidence to support that exergaming was a gateway to behavior change [35]. Identifying significant enjoyment predictors, game features and under what conditions mobile games can enhance behavior change are necessary.

There are some tricks and barriers that may limit the effects of Pokémon Go on players’ physical activity. Players may cheat by taking slow-moving transportations like buses and trams to earn distances. Instead of walking, those who own a car can drive to Pokéstops. Other issues such as battery drain, running out of mobile data, GPS problems with inaccurate location, and body discomforts like leg tiredness, eye strain and neck pain, etc., can limit people’s playing time. A common limitation in using smartphone games in health promotion, especially to the economically disadvantaged people, is the availabilities of a smartphone and mobile data. Elderly people and those with poor vision may also have difficulties in operating smartphones. Choosing budget smartphones with a large screen and a bigger keypad, and using economic data plans with slower data speed may help minimizing the problems.

The study population was limited to students in a local university and they are not representative to the general population. The time spent in walking/jogging activities and physical activity levels prior to playing the game was not studied because of the possibility of recall bias. The frequencies of and length of time in staying outdoors, walking and jogging, and other moderate and vigorous physical activities were self-reported by the respondents. There might be recall bias and the activity levels could also be over-reported because of social desirability effects. In future studies, the walking distances of players, ex-players and non-players need to be measured using distance measuring applications or pedometers while physical activity patterns can be recorded using accelerometers to obtain more precise data. Prior physical activities of players have to be recorded for comparison. Changes in BMI or body weight, and the extent of users replacing indoor activities with outdoor activities can also be studied to investigate the potential of incorporating Pokémon Go or similar AR games into exercise and weight management programs. The long-term impact of Pokémon Go on physical activity levels of the general population and motivators to sustain an active lifestyle can also be further explored.

## Conclusions

This study showed that players who were used to being sedentary benefited the most from Pokémon Go. Players who never or rarely stayed outdoors or walked/jogged before significantly stay outdoors more frequently as well as walking/jogging more often in order to play Pokémon Go. However, substantial impact on physical activity levels of players could not be identified. A possible alternative to promote physical activity is to engage people in using tools and technology they currently possess and are familiar with, such as mobile devices and games [12]. Developing a mobile health app is costly and the testing usually takes a long time. As user satisfaction and interest on new exergames are uncertain, using existing popular apps is another option. A well-rounded physical activity should include aerobic exercise and resistance training. Although Pokémon Go cannot be used to replace ordinary exercise as its benefits on cardio health are limited unless the players jog and walk briskly, it can be used as a starting point for sedentary people to begin an active lifestyle. The influence of Pokémon Go and other exergames on people’s health can provide insights to public health workers in using novel strategies in health promotion.
