# Erratum to: Influence of Pokémon Go on physical activity levels of university players: a cross-sectional study

**DOI:** 10.1186/s12942-017-0089-5

**Published:** 2017-04-26

**Authors:** Fiona Y. Wong

**Affiliations:** Faculty of Health and Social Sciences, The Hong Kong Polytechnic University, Kowloon, Hong Kong China

## Erratum

Unfortunately, the original version of this article [1] contained a few errors. The following sentence from the abstract: “The players spent a mean of 108.19 ± 158.21 min/week to walk/jog in order to play the game which is equivalent to burning 357 kcal/week for a 60-kg person walking a moderate pace.” should have been clearer. This has now been corrected to “**Players who never or rarely walked/jogged before** spent a mean of 108.19 ± 158.21 min/week to walk/jog in order to play the game which is equivalent to burning 357 kcal/week for a 60-kg person walking a moderate pace.”

Furthermore, the following sentence was also incorrect: “39.5% claimed that they were players (still playing Pokémon Go at the time of the survey), 30.9% claimed that they were ex-players (played before but had not played for at least 7 consecutive days), and the remaining 29.6% were non-players (never played the game) (Table 1).” This should have been “**37.7%** claimed that they were players (still playing Pokémon Go at the time of the survey), **27.6%** claimed that they were ex-players (played before but had not played for at least 7 consecutive days), and the remaining **34.6%** were non-players (never played the game) (Table 1).” The changes to the sentences are in boldface for clarity.
